# Case of Foreign Body External to the Knee-Joint

**Published:** 1872-05

**Authors:** T. Curtis Smith

**Affiliations:** Middleport, Ohio


### CASE OF FOREIGN BODY EXTERNAL TO THE KNEE-JOINT.

BY T. CURTIS SMITH, M. D., MIDDLEPORT, OHIO.

Mr. L—, æt 45, col., naturally robust, is now bent, constitution enfeebled, and health impaired. He complained of a difficulty with his right knee, which he referred to a movable body at that point. On examination, I discovered a movable body about an inch and a half in diameter, which imparted a hard sensation and slightly nodulated. It could be brought down over the patella, where it was only covered by the integument and superficial fascia. From this point it could be pushed up the thigh about five inches, gradually approaching the deeper structures as it was carried upwards, until it could no longer be felt. He stated that he had consulted several surgeons, but none had ventured an opinion concerning the nature of his trouble, and therefore he had not allowed any treatment. I gave him my opinion that it was only a loose foreign body, was not attached to anything, nor was it connected with the knee-joint, but entirely external to it, and therefore there would be no danger beyond that of a free incision attending its removal. He consented to have it removed, which I did by pushing it down over the patella, making a free incision over its whole diameter, vertically, then lifting one edge of it with the handle of the scalpel, it popped out on the floor. The incision was tightly closed by a firm adhesive plaster, and healed very kindly, only a very few drops of blood being observed, and no evidence of synovial fluid escaping. A rapid recovery ensued.

The body consisted of fat, cartilege, and at one small point was ossified. It was patella shaped, slightly nodulated and fifteen lines in diameter.

The old man's vaguery concerning it was, that the witches had put it in there or caused it to grow there as a punishment for doing some displeasure to their wishes.

This case is not reported as much for its great importance, practically, as for its rarity. Quite a number of cases of foreign bodies *in* the knee-joint, and their successful removal, have been reported, but I know of none that is exactly parallel with this one, where the body was external to the joint and capable of being so freely moved.

